# COVID-19 in dental care: What do we know?

**DOI:** 10.1080/20002297.2021.1957351

**Published:** 2021-07-29

**Authors:** Marek Chmielewski, Oliwia Załachowska, Weronika Rybakowska, Dominika Komandera, Agata Knura, Adrian Albert, Julia Kostanowicz, Katarzyna Garbacz

**Affiliations:** a Oral Microbiology Student Scientific Club, Medical Faculty, Medical University of Gdansk, Gdansk, Poland; b Department of Oral Microbiology, Medical Faculty, Medical University of Gdansk, Gdansk, Poland

**Keywords:** Dental care, SARS-COV-2, COVID- 19, oral cavity, triage

## Abstract

SARS-CoV-2 (severe acute respiratory syndrome coronavirus 2) is primarily transmitted by airborne droplets and its spread is favored by close human contact, thus the COVID-19 pandemic is the new challenge in dental practice. The oral cavity was considered as a SARS-CoV-2 reservoir, the viruses were detected in the saliva and periodontal pockets of infected persons. Therefore, aside from the most common symptoms, COVID-19 can manifest as lesions in the oral cavity. Due to the high risk of cross-contamination in the dental office, new precautionary measures were implemented in professional dental care to ensure safety for both, dental staff and patients. Given the dynamically changing situation, dental practitioners should follow local guidelines and implement them according to current needs and available resources. The key to success is to reduce the risk of cross-infection with SARS-CoV-2 at no cost to the good oral health of the population.

## Introduction

On 11 February 2020, the World Health Organization (WHO) announced a pandemic caused by the severe acute respiratory syndrome coronavirus 2 (SARS-CoV-2). Coronavirus disease 2019 (COVID-19) was first identified in Wuhan, China, in patients with atypical pneumonia [1].

SARS-CoV-2 belongs to the *Coronaviridae* (CoVs) family, the *Beta-coronavirus* genus. The name of the family is derived from the solar corona-like spikes located on the surface of the virion. CoVs are enveloped positive-single-stranded RNA viruses. The large RNA genome (27–32 kb) is associated with the N protein, forming a helical nucleocapsid. The envelope contains three structural proteins: membrane protein (M), spike protein (S) and envelope protein (E). Aside from the four structural proteins (S, E, M, and N), CoVs have 16 non-structural proteins (nsp 1–16) [2–4].

Transmission of SARS-CoV-2 may occur via a direct or indirect route. The virus is primarily transmitted with respiratory droplets and aerosol during coughing, sneezing and talking. SARS-CoV-2 enters the host’s body through the mucosal membranes of the mouth, nose and eyes. The transmission can occur during close contact between infected and uninfected persons. Although COVID-19 is primarily an airborne disease, the virus can also be transmitted through objects contaminated with body fluids, especially saliva [5,6].

COVID-19 may have various forms, from asymptomatic infection to the severe acute respiratory syndrome [7]. The most commonly occurring signs of the disease include fever > 38°C, coughing, sore throat and headache [8,9]. Shortness of breath, dyspnea and pneumonia are observed less often. Neurological symptoms, such as dizziness and impaired consciousness, are also reported in COVID-19 patients [10].

## The oral cavity as a potential reservoir of SARS-CoV-2

From the beginning of the COVID-19 pandemic, the spread of SARS-CoV-2 has been associated with microdroplets emitted from the oral cavity [11]. Increased moisture and temperature and hindered hygienic access to some regions make the oral cavity a perfect environment for virus survival [12]. The two possible oral reservoirs of SARS-CoV-2 are currently being studied: salivary glands and periodontal pockets.

SARS-CoV-2 can be detected in the saliva of infected patients. To produce adequate amounts of saliva, salivary glands utilize multiple molecules that bind to their specific receptors [5]. One of these receptors is the angiotensin-converting enzyme-2 (ACE-2) receptor. Notably, the same receptor is also used by SARS-CoV-2 to enter and infect the host’s cells [13]. One study demonstrated that the epithelial expression of the ACE-2 receptor in the salivary glands was one of the highest within the head and neck region [14]. The levels of the ACE-2 receptor in the salivary glands were shown to be similar as in the lungs or even higher, especially in minor glands. High ACE-2 receptor density in the salivary glands might be a cause of asymptomatic infections playing a pivotal role in SARS-CoV-2 transmission. Salivary glands are supposed to be a target for SARS-CoV-2 and a major source of the virus in the saliva. Unfortunately, we still do not have enough evidence regarding the excretion of the virus to the saliva, and hence, no ultimate conclusions can be made about the role of salivary glands infection in the etiopathogenesis of either prolonged or recurrent COVID-19 [15].

Periodontal pockets can be a favorable niche for SARS-CoV-2, where the virus can survive and be excreted with saliva or spread systemically via periodontal capillaries. Previous studies showed that some viruses, such as herpes simplex virus and Epstein-Barr virus, can survive in periodontal pockets for a long time [16]. SARS-CoV-2 might be another virus using periodontal pockets as a starting point for further spread. Colonization of periodontal pockets with some microorganisms, such as *Prevotella, Fusobacterium*, and *Porphyromonas*, was shown to negatively affect human health and lead to recurring infections if untreated [17]. Further, similar to SARS-CoV-2, those periodontal pathogens can be involved in the etiopathogenesis of respiratory infections [18].

Several potential mechanisms have been postulated to be involved in the promotion of pneumonia by oral bacteria [19]. The first of those mechanisms is the aspiration of oral microorganisms, such as *Prevotella, Fusobacterium* and streptococci, into the lower airways. Those microorganisms, involved in periodontal disease and tooth decay, can trigger the secretion of proinflammatory cytokines (IL-1, TNF), which may lead to inflammation or promote infection within the lungs [18,20]. A similar mechanism might also be involved in the pathogenesis of COVID-19. Another potential mechanism might be the activity of enzymes associated with periodontal disease; altering the properties of mucosal surfaces, those enzymes may promote adhesion and colonization by respiratory pathogens. Also, the secretion of periodontal disease-associated cytokines might lead to alterations of the respiratory epithelium, making the latter more prone to infection with respiratory pathogens [21]. Other comorbidities associated with periodontal disease include type 2 diabetes mellitus, Alzheimer’s disease, cardiovascular disorders, chronic obstructive pulmonary disease and sepsis. These conditions are postulated to increase the risk of severe complications and death from COVID-19 [22–25].

Some patients with COVID-19 require mechanical ventilation. This procedure may lead to bacterial colonization of the oropharynx due to impaired self-cleansing of the oral cavity associated with decreased salivary secretion. This may eventually result in ventilator-associated pneumonia (VAP), a condition that can increase the mortality of Intensive Care Unit patients by 30–70% [26]. Many studies showed that optimal oral care could significantly decrease the VAP risk [26] and facilitate the management of COVID-19 complications [27].

## Oral symptoms of COVID-19

Since the outbreak of COVID-19, multiple manifestations and outcomes of this viral disease have been described in various organs and systems (lungs, blood vessels, kidneys, brain, etc.) [28]. Many authors reported various oral symptoms in SARS-CoV-2-positive patients. One of the most common and distinctive signs of COVID-19 are ageusia and anosmia. While these manifestations are considered a neurological problem, the loss of taste is with no doubt connected with the function of the stomatognathic system. More than 60% of infected patients report the loss of taste during surveys and interviews [29]. Another frequent manifestation of COVID-19 is dry mouth. In one study, including more than 120 patients, 56.25% reported discomfort caused by saliva deficiency, and xerostomia was shown to correlate with ageusia [30].

A growing body of evidence suggests that SARS-CoV-2-positive patients may present with mucosal lesions in the oral cavity. Ulcers are one of the most frequently observed manifestations of SARS-CoV-2 infection. According to Brandão et al., aphthous-like ulcerations were found in seven out of eight (87.5%) patients, and 50% of individuals who tested positively for SARS-CoV-2 presented with necrotic-like ulcers. The latter type of ulcers seems to be more common in older and immunosuppressed patients, whereas younger persons are more likely to develop the aphthae [31]. Amorim dos Santos et al. described a case of a COVID-positive patient whose dorsum of the tongue was covered with yellowish pinpoint ulcers, similar to those observed in recurrent herpes simplex [32]. Furthermore, both Brandão et al. and Amorim dos Santos et al. reported about the presence of necrotic ulcers with a characteristic pattern typical for recurrent HSV-1 infection [31,32].

Corchuelo and Ulloa described a focal thrush lesion that coexisted with aphthous-like ulcerations [33]. A pseudomembranous lingual thrush, similar to those observed in candidiasis, was also reported by Rodríguez [34]. The presence of a white plaque on the tongue, raising suspicion of candidiasis, was also described by Dos Santos et al. However, microbiological examination of the tongue swab revealed *Saccharomyces cerevisiae*, rather than *Candida* spp [32]. Plaque-like changes on the tongue were also reported by nine out of 128 (7%) COVID-19 patients who participated in an online survey conducted by Biadsee [30].

COVID-19 patients also tend to present with other types of oral mucosal lesions, such as reddish maculae or petechiae. The presence of petechiae located on the anterior hard palate was reported by Brandão et al. [31], and Cruz Tapia et al. described a patient with reddish maculae, also located on the hard palate [35]. Moreover, Corchuelo and Ulloa published a case report of a female COVID-19 patient with petechiae on the inner surface of the lower lip [33].

Cruz Tapia et al. reported about two COVID-19 patients with angina bullosa hemorrhagic-like (ABH-like) lesions located on the tongue and hard palate, respectively [35]. Small hemorrhagic ulcers were also found in two patients described by Brandão et al. [31]. Corchuelo and Ulloa described an atypical oral manifestation of COVID-19: the gingiva in a fair-skinned patient changed its color to brownish [33]. To the best of our knowledge, this the only published case report of such a sign of COVID-19.

Several hypotheses exist regarding the etiology of lesions found in the oral cavity of COVID-19 patients. According to the first hypothesis, acinar cells infected by SARS-CoV-2 might produce less saliva. As already mentioned, some SARS-CoV-2-positive patients can present with dry mouth and taste disorders due to hyposalivation [31,36]. Given that saliva is a crucial factor in the perception of taste, and taste buds’ receptors can be activated only by dissolved substances, hyposalivation may lead to dysgeusia [37]. As mentioned in the previous section, ACE-2 receptors are abundant in the oral epithelium. However, the density of ACE-2 receptor expression is area-specific, with more receptors found on the tongue than in buccal and gingival areas [38]. Importantly, saliva can also be the main reservoir of SARS-CoV-2 in the oral cavity [36]. Thus, local infection with the virus may trigger inflammation and necrosis with the formation of ulcers and aphthae, especially in the areas with high ACE-2 receptor density [31].

According to another theory, some of the oral manifestations of COVID-19 might be a consequence of opportunistic infections [32,39]. The cytokine storm associated with SARS-CoV-2 infection leads to over-activation of T-cells [28,40], which in a longer perspective may result in the depletion of those cells and immune deficiency [40]. Patients with lymphopenia and persons treated with broad-spectrum antibiotics were previously shown to be more prone to oropharyngeal candidiasis [39]. This observation may explain why some COVID-19 patients present with thrush lesions or oral manifestations typical for recurrent herpes simplex or fungal infections. Also, the case of gingival discoloration described earlier in this section might be linked to the cytokine storm; gingival hyperpigmentation might be a consequence of enhanced melanogenesis, indirectly stimulated by cytokines, such as TNF-α and IL-1α [33].

Some of the oral manifestations of COVID-19 might also be associated with thrombocytopenia. In a Chinese study, thrombocytopenia was found in 36.2% of 1,099 COVID-19 patients [40]. Patients with thrombocytopenia are known to be more prone to the formation of ABH-like lesions and petechiae [41]. According to some authors, the oral changes present in patients with COVID-19 might also be a consequence of vasculitis or disseminated intravascular coagulation (DIC) [42].

## Triage of dental patients during the COVID-19 pandemic

Due to the specific conditions of a dental practice, both staff and patients are at increased risk of cross-infection with SARS-CoV-2; the risk is inherent to close contact between these two groups, as well as to the exposure to saliva, aerosol and other secretions [43,44]. However, despite the pandemic, dental care cannot be neglected given the considerable impact of oral health on general health. Given the dynamically changing situation, dental practitioners should follow and be updated on local infection control guidelines and implement them according to current needs and available resources. Therefore, dentists are advised to perform their duties in a way that limits the dissemination of SARS-CoV-2 and ensures the safety of both dental staff and patients. Accurate triage is a prerequisite of achieving these objectives [45].

Proper communication is the first control measure to be undertaken even before seeing the patient [46]. Gurzawska-Comis et al. recommend triaging patients via telephone conversation or video conference. All patients with symptoms raising suspicion of COVID-19 should use a teledentistry consultation before visiting the dental clinic [46]. Apart from identifying suspected or confirmed COVID-19 cases beforehand, another purpose of the triage is to assess the urgency of each referral [47,48]. In Italy, it is compulsory to carry out a telephone triage to verify whether a patient’s visit to the clinic is essential [49]. In Slovenia, smartphone-taken pictures and videos of the affected area are used during the triage [48] to establish a more accurate diagnosis and to ultimately decide whether the patient should visit the clinic or not [46].

During the triage, it is assessed how likely is the patient to be infected with SARS-CoV-2 and whether he/she belongs to a high-risk group of the infection [50]. According to Gurzawska-Comis et al., persons who had contact with SARS-CoV-2-positive patients or visited any high-risk region according to the WHO in the last 14 days are at very high risk of being infected, even despite the lack of flu-like symptoms. Also, patients with a negative epidemiological history, albeit presenting with flu-like symptoms, should be classified into the high-risk group. Whenever possible, appointments with infected or potentially infected patients should be postponed optimally for two weeks [48,51]. During this period, the patients should be managed remotely via teledentistry. The use of a three A scheme, i.e. Advice, Analgesia and Antibiotics (Antimicrobials), is advised for remote dental care [44,48,52–54]. If on admission to the clinic the patient presents with fever (> 38°C or > 37.5°C according to Izzetti et al. [49], the appointment should be postponed for at least 2–3 weeks, except for real emergencies. If the visit cannot be delayed, all preventive measures recommended for the management of high-risk patients should be executed [48,52,55].

Regarding the urgency of dental treatment, the referrals can be classified as emergent, urgent or elective [46]. The emergent cases require on-site management given the increased risk of the patient’s mortality. This group of cases includes uncontrolled hemorrhage, cellulitis or diffuse bacterial infection, and facial bone trauma with the risk of airway obstruction/damage [46,51,53].

According to Gurzawska-Comis et al. [44], patients’ needs should be categorized as urgent, handled as soon as possible (ASAP) or postponed (no urgency). The first group includes cases that require prompt treatment (within a 24-hour timeframe) but, unlike the emergencies, are not life-threatening, i.e. abscess drainage, acute dental pain when analgesics are no longer sufficient, pericoronitis, alveolitis, facial or dental trauma with avulsion or luxation, risk of airway obstruction (due to loose restoration), suspected malignancy, adjustments of orthosis and prosthesis that cause pain, changing intracanal medication, and removal of extensive dental caries or restorations that cause pain. The needs to be handled ASAP, i.e. within the next seven days, include the extraction of teeth due to chronic pain, cyst causing paresthesia/pain, temporary filling/loss of restoration and pain from broken ortho-appliance [44,46,50,56].

All elective extractions and elective restorative/periodontal/orthodontic treatments should be postponed. According to Dacic et al., all those procedures can be safely delayed for approximately 30 days [52].

It should be stressed that susceptible or vulnerable patients should be prioritized. According to Shamsoddin et al., this group includes patients with chronic diseases and malignancies, pregnant women, nursing home residents and prisoners. All these groups require unlimited access to dental care as their overall health condition is not infrequently worse than in the general population [45]. The key to success is to reduce the risk of cross-infection with SARS-CoV-2 at no cost to the good oral health of the population.

## Prevention of COVID-19 transmission in the dental practice

As COVID-19 is an airborne disease, its prevention is based primarily on limiting the air transmission of SARS-CoV-2. Dentistry is considered one of the most SARS-CoV-2-exposed professions, which warrants the implementation of strict preventive measures in everyday practice [57].

The first recommended form of prevention is the triage of dental patients, described in detail in the section above. In the case of patients visiting the clinic, consecutive appointments should be scheduled with at least 15-minute breaks in between to allow for proper disinfection of the office and avoid patient-to-patient contact in the waiting room. No reading materials should be displayed in the waiting room as they may serve as a vector for the virus spread. Whenever two or more patients are present in the waiting room, they should be seated at a 1–2 meter distance from one another. The dentist and the assistant, both wearing appropriate protective gears, should be present in the room before the patient enters [57]. After entering the office, the patient should be immediately directed to the dentist’s chair. Protective gears for the dental staff include disposable working caps, disposable surgical FFP2 masks (in the case of a patient with suspected SARS-CoV-2 infection, an FFP3 mask is recommended by the WHO) [58,59], standard working uniform, protective goggles or face shield and disposable nitrile or latex gloves. The disposables should be changed after each patient to reduce the risk of transferring the virus from the patient to the dentist or vice versa [26]. However, the recommendations on protective equipment or breaks between patients may differ locally or depending on the treatment of symptomatic or asymptomatic patients, or suspected COVID-19.

The patient should take off the mask no earlier than before the beginning of the dental examination and put it back immediately after the last procedure [57]. Many dental procedures involve rotary dental and surgical instruments with water cooling, which generate a considerable amount of aerosol. The aerosol contains saliva droplets with bacteria, viruses and sometimes blood [60]. Thus, to reduce the number of microbes released with the aerosol, some authors suggest rinsing the mouth with a chlorhexidine digluconate (CHX) solution before the beginning of dental treatment. It is suggested to use 15 ml of 0.12% CHX once to reduce the risk of COVID-19 transmission for up to 2 hours [61]. If the chlorhexidine is unavailable or contraindicated in a given patient, it can be replaced with 1% hydrogen peroxide. Coronaviruses were shown to be inactivated by 0.5% hydrogen peroxide; concentrations greater than 1% are not recommended as they may negatively affect hard and soft tissues, leading to complications, and at concentrations below 1% hydrogen peroxide is inactivated by salivary catalase [60]. The results of some recent studies suggest that also 0.23% povidone-iodine (PVP-I) and 0.05% cetylpyridinium chloride (CPC) mouthwashes could be effective against SARS-CoV-2 [62,63]. If their effectiveness against SARS-CoV-2 is confirmed in a clinical setting, they could be considered an alternative mouthwashes in patients who develop irritations and discoloration after CHX use [63].

Before examining the patient, the dentist needs to disinfect their hands. WHO recommends using alcohol-based rubs (60–70%) as they are known to be highly effective against the enveloped coronaviruses. Ethanol, the most common component of alcohol-based disinfectant gels, inactivates bacteria and viruses, including SARS-CoV-2. Although little is known about the frequency of hand contamination with SARS-CoV-2 and the viral load on hands after contact with contaminated surfaces, WHO recommends applying alcohol-based rubs for effective hand decontamination [16]. Other potentially applicable, albeit less effective hand disinfectants include propanol, chlorhexidine digluconate and benzalkonium chloride solutions [64]. Only after appropriate hygienization of hands, in accordance with the Ayliffe scheme, the dentist can wear disposable gloves and proceed with the examination and treatment.

Another preventive measure that should be considered is the use of saliva ejectors. These devices were shown to reduce the production and spread of saliva droplets and aerosols [22]. During restorative or endodontic treatment, the use of a rubber dam is recommended as a barrier limiting the contact with the patient’s saliva [27]. Samaranayake et al. observed a significant decrease in aerosol production during the use of the rubber dam. Application of the dam together with other protective measures, namely disinfectant mouthwash, HVE filter suction and personal protective gear, was shown to significantly reduce the risk of infection with SARS-CoV-2 contained in the aerosol [65].

After the patient leaves the office, surface disinfection should be carried out. SARS-CoV-2 contained in aerosol particles is typically virulent for up to 3 hours, but it may survive up to 72 hours on some plastic or metal surfaces. Thus, WHO recommends alcohol-based surface disinfection or with a 5% sodium hypochlorite solution diluted with water in a 1:100 ratio. The solution should be applied on the surfaces for at least 10 minutes [66].

Another vital aspect to be considered is the decontamination of teeth impressions. The impressions should be decontaminated with 1% sodium hypochlorite. The protocol of decontamination varies depending on the material used for the impression. Alginate impressions should be kept in the sodium hypochlorite solution for 10 minutes and then thoroughly washed with water and transferred to the dental laboratory in moist conditions. Meanwhile, the impressions made of silicones should be kept in sodium hypochlorite for approximately 15–20 minutes [67].

It is also advised to ventilate the office after each patient left. If the office is equipped with an air conditioning system, the device should have HEPA filters, which need to be replaced regularly. If the HEPA filter has not been replaced for an extended period, it may become a source of microorganisms and constitute a health hazard [58,59].

In summary, this review summarizes the current information on the oral cavity as a potential reservoir of SARS-CoV-2, oral manifestations of COVID-19 and their possible background, as well as the essential recommendations against COVID-19 in dental care.
